# Supplementary material for: Exploring risk factors associated with catastrophic health expenditure on diarrhea management in Karachi, Pakistan, 2022-2024
Source: PLOS Glob Public Health. 2026 Jul 15;6(7):e0005391. doi: 10.1371/journal.pgph.0005391 (PMC13372140; doi:10.1371/journal.pgph.0005391)
Supplement: S1 Table — (DOCX) [file pgph.0005391.s001.docx]

**S1 Table: Sensitivity analysis of factors associated with catastrophic health expenditure on diarrhea care seeking among households of children aged 6-35 months in EFGH Pakistan Site, 2022-2024**

|  | Catastrophic spending threshold 8% | | Catastrophic spending threshold 5% | |
| --- | --- | --- | --- | --- |
|  | Unadjusted RR estimate (95% CI) | p-value | Unadjusted RR estimate (95% CI) | p-value |
| **Facility attributes** | | | | |
| **Facility ownership** | | | | |
| Private | Ref | Ref | Ref | Ref |
| Public | 0.44 (0.09-2.07) | 0.301 | 0.98 (0.33-2.92) | 0.976 |
| **Facility level** | | | | |
| Primary | Ref | Ref | Ref | Ref |
| Secondary | 0.29 (0.04-2.28) | 0.237 | 0.25 (0.03-2) | 0.193 |
| Tertiary | 0.98 (0.12-7.77) | 0.985 | 3.49 (1.09-11.15) | 0.035 |
| **Child demographics** |  |  |  |  |
| **Child sex** | | | | |
| Female | Ref | Ref | Ref | Ref |
| Male | 3.52 (0.75-16.52) | 0.110 | 1.17 (0.41-3.36) | 0.766 |
| **Child age** | | | | |
| 6-11 months | Ref | Ref | Ref | Ref |
| 12-23 months | 0.88 (0.24-3.24) | 0.842 | 0.7 (0.23-2.16) | 0.535 |
| 24-35 months | 0.38 (0.04-3.36) | 0.382 | 0.5 (0.1-2.48) | 0.398 |
| **Prior careseeking** | | | | |
| No | Ref | Ref | Ref | Ref |
| Yes | 3.05 (0.8-11.63) | 0.103 | 1.94 (0.55-6.85) | 0.304 |
| **Pathogen** | | | | |
| **Shigella** | | | | |
| Non-Shigella | Ref | Ref | Ref | Ref |
| Shigella | 0.83 (0.17-3.97) | 0.814 | 1.29 (0.4-4.16) | 0.671 |
| **Rotavirus** | | | | |
| Non-rotavirus | Ref | Ref | Ref | Ref |
| Rotavirus | 2.67 (0.57-12.43) | 0.211 | 1.78 (0.4-7.86) | 0.447 |
| **Adenovirus** | | | | |
| Non-adenovirus | Ref | Ref | Ref | Ref |
| Adenovirus | 0 (0-0) | 0.000 | 2.42 (0.32-18.2) | 0.392 |
| **Cryptosporidium** | | | | |
| Non- cryptosporidium | Ref | Ref | Ref | Ref |
| Cryptosporidium | 6.07 (1.29-28.61) | **0.023** | 3.86 (0.88-17.06) | 0.074 |
| **Enterotoxigenic Escherichia coli (ETEC)** | | | | |
| Non- Enterotoxigenic Escherichia coli (ETEC) | Ref | Ref | Ref | Ref |
| Enterotoxigenic Escherichia coli (ETEC) | 0.9 (0.11-7.17) | 0.921 | 1.31 (0.29-5.87) | 0.723 |
| **Child health characteristics** | | | | |
| **Chronic comorbidity** | | | | |
| No | Ref | Ref | Ref | Ref |
| Yes | 0 (0-0) | 0.000 | 6.9 (1.61-29.62) | **0.009** |
| **Acute comorbidity** | | | | |
| No | Ref | Ref | Ref | Ref |
| Yes | 1.5 (0.42-5.28) | 0.530 | 1.25 (0.42-3.7) | 0.690 |
| **Diarrhea severity** | | | | |
| Mild | Ref | Ref | Ref | Ref |
| Moderate | 1.85 (0.17-20.29) | 0.616 | 3.7 (0.52-26.1) | 0.190 |
| Severe | 11.12 (2.32-53.26) | **0.003** | 15.89 (3.5-72.13) | **0.000** |
| **Wasting** | | | | |
| None | Ref | Ref | Ref | Ref |
| Moderate | 3.63 (0.82-16.14) | 0.090 | 2.28 (0.7-7.41) | 0.172 |
| Severe | 6.45 (1.32-31.66) | **0.022** | 3.23 (0.82-12.79) | 0.094 |
| **Stunting** | | | | |
| Not Stunned | Ref | Ref | Ref | Ref |
| Stunted | 0.39 (0.08-1.83) | 0.233 | 0.26 (0.06-1.16) | **0.077** |
| **Underweight** | | | | |
| None | Ref | Ref | Ref | Ref |
| Moderate | 3.59 (0.86-14.92) | 0.079 | 1.54 (0.49-4.82) | 0.458 |
| Severe | 2.34 (0.39-13.91) | 0.351 | 1 (0.21-4.8) | 0.996 |
| **Household and caregiver characteristics** | | | | |
| **Caregiver age (years)** | 0.91 (0.81-1.03) | 0.147 | 0.94 (0.85-1.03) | 0.204 |
| **Caregiver work** | | | | |
| Not Working | Ref | Ref | Ref | Ref |
| Working | 3.77 (0.81-17.5) | 0.091 | 2.51 (0.57-11.07) | 0.224 |
| **Mother education** | | | | |
| None | Ref | Ref | Ref | Ref |
| Some or all primary school | 1.55 (0.35-6.88) | 0.565 | 1.55 (0.35-6.88) | 0.565 |
| Some or all secondary school | 0.44 (0.07-2.61) | 0.365 | 1.31 (0.33-5.21) | 0.702 |
| Koranic school only | 3.95 (0.42-37.01) | 0.229 | 3.95 (0.42-37) | 0.229 |
| **Father education** | | | | |
| None | Ref | Ref | Ref | Ref |
| Some or all primary school | 1.37 (0.28-6.74) | 0.699 | 1.83 (0.41-8.1) | 0.428 |
| Some or all secondary school | 0.63 (0.13-3.11) | 0.572 | 1.26 (0.32-5.01) | 0.744 |
| Koranic school only | 7.69 (0.84-70.36) | 0.071 | 7.69 (0.84-70.35) | 0.071 |
| **Number of children <5 in household** | 0.45 (0.18-1.14) | 0.093 | 0.56 (0.28-1.12) | 0.101 |
| **Travel time to clinic** | | | | |
| <30 min | Ref | Ref | Ref | Ref |
| 30 min or more | 0.84 (0.18-3.95) | 0.827 | 0.92 (0.26-3.28) | 0.896 |
| **Wealth quintile** | | | | |
| Quintile 1 (fewest assets) | Ref | Ref | Ref | Ref |
| Quintile 2 | 0.39 (0.08-1.98) | 0.253 | 0.77 (0.21-2.86) | 0.697 |
| Quintile 3 | 0.39 (0.05-3.34) | 0.393 | 0.78 (0.15-3.99) | 0.768 |
| Quintile 4 | 1.08 (0.21-5.51) | 0.928 | 1.08 (0.21-5.51) | 0.929 |
| Quintile 5 (most assets) | 0 (0-0) | 0.000 | 1.48 (0.18-12.45) | 0.720 |

CI: 95% confidence interval, Significant RR is presented in bold

^1^All variables were adjusted for diarrhea severity
